# Shock subtypes by left ventricular ejection fraction following out-of-hospital cardiac arrest

**DOI:** 10.1186/s13054-018-2078-x

**Published:** 2018-06-15

**Authors:** Ryan J. Anderson, Sayuri P. Jinadasa, Leeyen Hsu, Tiffany Bita Ghafouri, Sanjeev Tyagi, Jisha Joshua, Ariel Mueller, Daniel Talmor, Rebecca E. Sell, Jeremy R. Beitler

**Affiliations:** 1 Division of Pulmonary and Critical Care Medicine, Stanford University, Stanford, CA USA; 2 Department of Anesthesia, Critical Care, and Pain Medicine, Beth Israel Deaconess Medical Center and Harvard Medical School, Boston, MA USA; 3 Department of Medicine, Weill Cornell Medical College, New York, NY USA; 4 Department of Medicine, University of California San Diego, San Diego, CA USA; 5 Division of Pulmonary and Critical Care Medicine, University of California San Diego, San Diego, CA USA; 6 Center for Acute Respiratory Failure, Division of Pulmonary, Allergy, and Critical Care Medicine, Columbia University College of Physicians & Surgeons, 622 W. 168th Street, 8E101, New York, NY 10032 USA

**Keywords:** Cardiac arrest, Shock, Cardiogenic shock, Distributive shock, Reperfusion injury, Systemic inflammatory response syndrome

## Abstract

**Background:**

Post-resuscitation hemodynamic instability following out-of-hospital cardiac arrest (OHCA) may occur from myocardial dysfunction underlying cardiogenic shock and/or inflammation-mediated distributive shock. Distinguishing the predominant shock subtype with widely available clinical metrics may have prognostic and therapeutic value.

**Methods:**

A two-hospital cohort was assembled of patients in shock following OHCA. Left ventricular ejection fraction (LVEF) was assessed via echocardiography or cardiac ventriculography within 1 day post arrest and used to delineate shock physiology. The study evaluated whether higher LVEF, indicating distributive-predominant shock physiology, was associated with neurocognitive outcome (primary endpoint), survival, and duration of multiple organ failures. The study also investigated whether volume resuscitation exhibited a subtype-specific association with outcome.

**Results:**

Of 162 patients with post-resuscitation shock, 48% had normal LVEF (> 40%), consistent with distributive shock physiology. Higher LVEF was associated with less favorable neurocognitive outcome (OR 0.74, 95% CI 0.58–0.94 per 10% increase in LVEF; *p* = 0.01). Higher LVEF also was associated with worse survival (OR 0.81, 95% CI 0.67–0.97; *p* = 0.02) and fewer organ failure-free days (β = – 0.67, 95% CI – 1.28 to − 0.06; *p* = 0.03). Only 51% of patients received a volume challenge of at least 30 ml/kg body weight in the first 6 h post arrest, and the volume received did not differ by LVEF. Greater volume resuscitation in the first 6 h post arrest was associated with favorable neurocognitive outcome (OR 1.59, 95% CI 0.99–2.55 per liter; *p* = 0.03) and survival (OR 1.44, 95% CI 1.02–2.04; *p* = 0.02) among patients with normal LVEF but not low LVEF.

**Conclusions:**

In post-resuscitation shock, higher LVEF—indicating distributive shock physiology—was associated with less favorable neurocognitive outcome, fewer days without organ failure, and higher mortality. Greater early volume resuscitation was associated with more favorable neurocognitive outcome and survival in patients with this shock subtype. Additional studies with repeated measures of complementary hemodynamic parameters are warranted to validate the clinical utility for subtyping post-resuscitation shock.

**Electronic supplementary material:**

The online version of this article (10.1186/s13054-018-2078-x) contains supplementary material, which is available to authorized users.

## Background

Post-resuscitation circulatory shock occurs in most patients admitted after out-of-hospital cardiac arrest (OHCA) and is a primary contributor to subsequent mortality [1–3]. Optimal hemodynamic management of post-resuscitation shock is complicated by the myriad pathophysiological processes involved—which together are termed post-cardiac arrest syndrome (PCAS) [4–7].

Post-resuscitation global myocardial stunning can cause transient pump failure lasting several hours [1, 8–10], and is thought to result from a combination of oxidative stress, microthrombi formation, adrenergic excess, cytokine release, and myocardial ischemia–reperfusion injury [7, 11–13]. When present, chronic systolic heart failure and acute coronary syndrome also may contribute to post-arrest cardiogenic shock [14, 15].

At the same time, global ischemia–reperfusion injury may precipitate systemic vasodilation. Associated systemic inflammatory response, endothelial injury, capillary leak, impaired vasoregulation, end-organ microvascular thrombi, and adrenal suppression share many similarities with septic shock [7, 16]. Concomitant infection also appears to be common and may contribute further to distributive shock [17].

Distinguishing the relative contributions of pump failure (cardiogenic shock) and vasodilation (distributive shock) from post-resuscitation shock may have important implications for personalizing hemodynamic management, particularly if done readily at the bedside. Therefore, the present study investigated whether early assessment of left ventricular ejection fraction (LVEF) could identify subtypes of shock that have potential prognostic and therapeutic relevance, including specifically whether the association between early intravascular volume resuscitation and clinical outcome differed by shock subtype.

## Methods

This study was approved by the hospitals’ institutional review boards with waiver of consent. See Additional file 1 for further details on methods.

### Study population

**Fig. 1:**
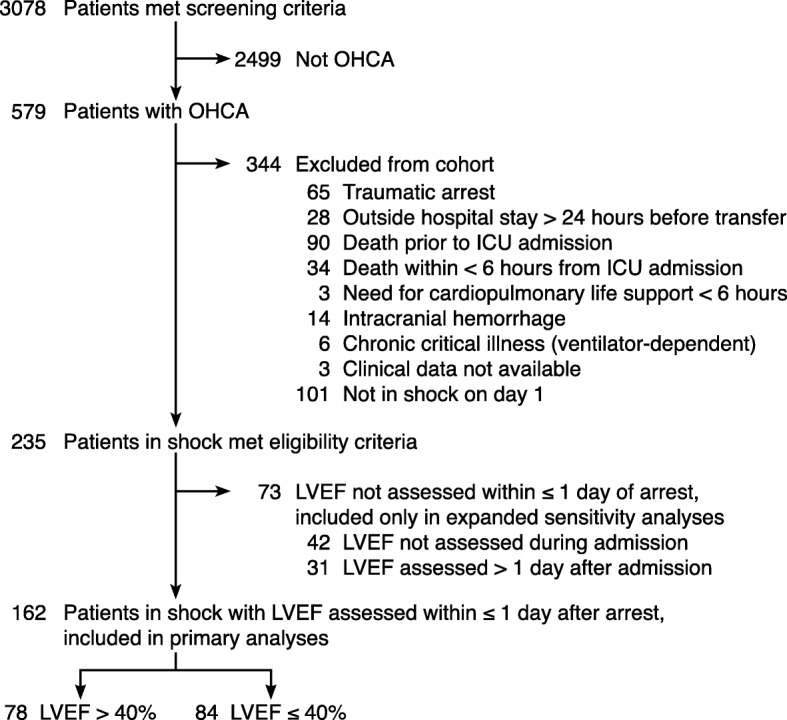
Study flow diagram. OHCA out-of-hospital cardiac arrest, ICU intensive care unit, LVEF left ventricular ejection fraction

A two-hospital retrospective cohort was assembled using a previously validated approach for identifying OHCA admissions [18, 19]. Pertinent medical records between 2008 and 2014 were screened using relevant billing codes [18] and reviewed manually by physician investigators to confirm eligibility. Included were adults aged ≥18 years in shock following nontraumatic OHCA who required advanced life support (vasopressors and/or mechanical ventilation) for at least the first 6 h of admission but survived over that time. Shock was defined as systolic blood pressure ≤ 90 mmHg or vasopressor administration, criteria adopted from the Brussels Conference on Clinical Trials for the Treatment of Sepsis [20] and used elsewhere in prominent ICU clinical trials networks [21]. Patients who met this definition of shock at any time within the first 24 h of ICU admission were considered to have shock on day 1. LVEF assessment within 1 day post arrest was required for inclusion in the main study cohort to reflect early pathophysiology in light of post-arrest myocardial dysfunction that may evolve over successive days [1]. Exclusions are defined in Fig. 1.

A second, expanded cohort was created for sensitivity analyses, detailed in the following, by including patients meeting all eligibility criteria irrespective of whether LVEF was assessed within 1 day post arrest. In this expanded sensitivity cohort, LVEF assessment was considered at any time during admission; patients without LVEF assessment during admission were assumed to have normal LVEF. Such handling of LVEF in the expanded sensitivity cohort was based on the following reasoning: if LVEF was depressed later during the hospitalization, it likely also was depressed at ICU admission; and pretest probability guides the use of echocardiography and ventriculography, such that if clinicians had little suspicion for depressed LVEF clinically, then LVEF was less likely to be measured.

### Left ventricular ejection fraction

LVEF was ascertained from either echocardiogram or left ventriculogram reports, whichever was performed on the earliest hospital day post arrest. If LVEF was reported from both methods on the same calendar day, the value from the left ventriculogram was used. LVEF > 40% was considered normal for this study, based on international consensus guidelines that define systolic heart failure below this threshold [22, 23].

### Primary outcome

The primary outcome, specified a priori for consistency with existing literature [24–28], was favorable neurocognitive outcome at hospital discharge, defined as Cerebral Performance Category (CPC) 1 or 2 (see Additional file 1) [29, 30]. CPC was determined independently by two study physicians blinded to LVEF, hemodynamic/resuscitation data, and other baseline illness severity measures as described previously [19], with discordant ratings resolved by consensus.

### Secondary outcomes

Secondary outcomes included shock-free days, ventilator-free days, renal, hepatic, and coagulation failure-free days, as well as days free from any of the aforementioned organ failures through day 28 (see Additional file 1). ICU-free days and hospital-free days were also evaluated.

### Statistics

For all analyses, a two-sided α threshold of 0.05 was used for statistical significance.

#### Main analyses of clinical outcomes

LVEF was entered as a continuous variable for all main analyses. This approach was chosen, rather than dichotomizing LVEF, to allow for potential differences in prognostic relevance within the range of systolic heart failure [31]. Odds ratios and model effect estimates are reported per increase of 10% in LVEF (e.g., from 30% to 40%). In the prespecified statistical plan, the main analyses of all clinical endpoints were adjusted for APACHE II score to account for differences in illness severity on admission. Linearity between LVEF and the primary outcome on the log-scale was assessed by inserting higher-order quadratic and cubic terms for LVEF in the model and testing for statistical significance, and by recoding LVEF as a log-transformed variable and comparing model fit (*c*-statistic) to the model with nontransformed LVEF. For graphical presentation, analysis of the main outcome was repeated using a Cox model and plotted as the APACHE II score-adjusted cumulative incidence for discharge with favorable neurocognitive outcome according to normal vs low LVEF. The proportional hazards assumption was confirmed via a second Cox model that included an interaction term for LVEF with time; a nonsignificant interaction coefficient supported proportionality.

#### Sensitivity analyses for primary outcome

Multiple sensitivity analyses were performed for the primary outcome, favorable neurocognitive outcome, to ensure findings were not dependent on the method of covariate adjustment or handling of the main predictor or outcome variables (see Additional file 1).

#### Subgroup analysis by initial rhythm

Initial arrest rhythm was not included as a covariate due to concern that the shock subtype may be downstream of the causal pathway and hence partially mediate the effect of the arrest rhythm. Thus, to ensure that LVEF was not simply a marker of the initial arrest rhythm, subgroup analyses were performed separately for patients with shockable and nonshockable rhythm, adjusting for APACHE II score. To address limited statistical power from small subgroup sizes, these analyses were repeated using the aforementioned expanded sensitivity cohort with dichotomized LVEF.

#### Post-hoc analysis for residual confounding

To ensure LVEF was not simply a marker of early differences in hemodynamic stability, additional models were developed evaluating whether LVEF predicted mean arterial pressure or vasopressor dose (norepinephrine equivalent) either at baseline or as a time-weighted average over the first 48 h. The association between LVEF and therapeutic hypothermia also was evaluated, owing to concern for possible collinearity between LVEF and arrest rhythm, since hypothermia is most often applied for patients with shockable rhythm.

#### Volume resuscitation and clinical outcomes

To evaluate the subtype-specific association of volume resuscitation at 6 and 24 h with favorable neurocognitive outcome and survival, separate logistic models were constructed for patients with normal and low LVEF, adjusting for APACHE II score, baseline vasopressor dose (norepinephrine equivalent), and arrest rhythm, covariates selected a priori for clinical relevance.

## Results

### Characteristics of study population

One hundred and sixty-two patients met the eligibility criteria (Fig. 1) and were included in all analyses. LVEF was assessed via echocardiogram in 154 patients and by left ventriculogram in eight patients. An additional 73 patients met all eligibility criteria except for an LVEF assessment within 1 calendar day of arrest and were included only in the sensitivity analyses as noted; their baseline characteristics were comparable to those with normal LVEF (> 40%) (Table 1).

**Table 1:** Baseline characteristics for patients in shock according to LVEF assessed within 1 day after arrest

Patient characteristic	LVEF > 40% (*n* = 78)	LVEF ≤ 40% (*n* = 84)	*p* value	Initial LVEF unknown^a^ (*n* = 73)
Age (years)	63 ± 15	64 ± 15	0.73	64 ± 20
Female	26 (33%)	20 (24%)	0.22	27 (37%)
Prior medical history				
Coronary disease	18 (23%)	34 (40%)	0.02	18 (25%)^b^
Congestive heart failure	12 (15%)	34 (40%)	< 0.01	17 (23%)^b^
Chronic pulmonary disease	12 (15%)	11 (13%)	0.82	17 (23%)
Arrest characteristics				
Witnessed arrest	56 (72%)	68 (81%)	0.20	48 (66%)^b^
Bystander CPR	41 (53%)	50 (60%)	0.43	37 (51%)
Time from collapse to CPR initiation (min)	2 (1–7)	2 (0–6)	0.97	2 (0–5)
Duration of CPR before sustained ROSC (min)	15 (10–38)	18 (9–30)	0.65	20 (11.5–42)
Initial rhythm VT/ VF	25 (32%)	61 (73%)	< 0.01	22 (30%)^b,c^
Comatose after ROSC	72 (92%)	80 (95%)	0.52	68 (93%)
Therapeutic hypothermia after ROSC	59 (76%)	75 (89%)	0.02	51 (70%)^b,c^
Markers of cardiac injury				
Peak troponin in first 24 h (ng/ml)	0.3 (0.1–1.0)	0.9 (0.3–2.7)	< 0.01	0.3 (0.1–0.9)^b,c^
ST-elevation myocardial infarction	8 (10%)	23 (27%)	.01	6 (8%)^b,c^
Coronary angiography during hospitalization	18 (23%)	45 (54%)	< 0.01	13 (18%)^c^
Coronary stent placed during hospitalization	7 (9%)	20 (24%)	0.01	6 (8%)^b^
Intraaortic balloon pump during hospitalization	3 (4%)	14 (17%)	< 0.01	6 (8%)
LVEF (%) within ≤ 1 day after arrest	59 ± 10	26 ± 9	< 0.01	N/A
Markers of systemic illness severity				
APACHE II score	35 ± 6	36 ± 6	0.09	35 ± 7
SOFA on day 1	12 ± 3	12 ± 3	0.67	12 ± 3
Number of organ failures on day 1^d^	3 ± 1	3 ± 1	0.97	3 ± 1
Initial vasopressor dose, norepinephrine equivalent (μg/min)	7.5 (2.5–20.1)	10.1 (2.6–21.5)	0.45	10.4 (3.5–21.7)
Initial lactate (mmol/L)	5.8 ± 4.7	4.1 ± 2.8	0.01	6.6 ± 5.2^b,c^
Peak lactate in first 24 h (mmol/L)	5.9 ± 4.7	4.6 ± 3.1	0.04	7.6 ± 6.1^b,c^
Initial respiratory characteristics				
Tidal volume (ml/kg PBW)	8.0 ± 1.9	8.1 ± 1.8	0.79	8.0 ± 1.3
PEEP (cmH_2_O)	5 (5–8)	5 (5–10)	0.14	5 (5–5)^b,c^
Peak inspiratory pressure (cmH_2_O)	27 ± 8	26 ± 8	0.75	27 ± 9
FiO_2_	100 (80–100)	100 (60–100)	0.40	100 (60–100)
pH	7.22 ± 0.19	7.23 ± 0.15	0.53	7.18 ± 0.20
PaCO_2_ (mmHg)	44 (39–62)	47 (37–56)	0.66	45 (37–57.5)
PaO_2_ (mmHg)	212 ± 128	202 ± 130	0.62	229 ± 167
PaO_2_:FiO_2_	237 ± 141	237 ± 148	0.99	258 ± 174
Volume resuscitation in first 6 h (L)	2.9 ± 1.8	2.7 ± 1.7	0.50	2.9 ± 2.0
Volume challenge ≥ 30 ml/kg in first 6 h	42 (54%)	41 (49%)	0.53	37 (51%)
Volume resuscitation in first 24 h (L)	6.1 ± 3.5	5.6 ± 3.1	0.34	6.0 ± 3.0

Descriptive statistics shown as mean ± standard deviation, median (interquartile range), or number (%) and compared with *t*, Wilcoxon rank-sum, or chi-square tests as appropriate

*LVEF* left ventricular ejection fraction, *CPR* cardiopulmonary resuscitation, *ROSC* return of spontaneous circulation, *VT* ventricular tachycardia, *VF* ventricular fibrillation, *N/A* not applicable, *APACHE II* Acute Physiology and Chronic Health Evaluation II, *SOFA* sequential organ failure assessment score, *PBW* predicted body weight, *PEEP* positive end-expiratory pressure, *FiO*_*2*_ fraction of inspired oxygen, *PaCO*_*2*_ partial pressure of carbon dioxide in arterial blood, *PaO*_*2*_ partial pressure of oxygen in arterial blood

^a^LVEF not assessed within ≤ 1 day post arrest, including 42 patients in whom LVEF was never assessed during admission and 31 patients in whom assessment occurred > 1 day after arrest

^b^*p* < 0.05 compared to patients with LVEF ≤ 40%. No values differed significantly compared to patients with LVEF > 40%

^c^*p* < 0.05 compared to all included patients with LVEF assessment within ≤ 1 day post arrest

^d^Cardiovascular failure defined as systolic blood pressure ≤ 90 mmHg or any vasopressor use. Respiratory failure defined by invasive mechanical ventilation. Coagulation, renal, and hepatic organ failures defined according to Brussels multiple organ dysfunction criteria

Eighty-three percent of included patients (135 of 162) meeting the shock criteria received continuous vasopressor and/or inotrope infusion at ICU admission, and 90% received continuous vasopressor/inotrope infusion within the first 48 h. Among patients meeting the shock criteria who were hypotensive but did not receive continuous vasopressor/inotrope infusion, initial lactate was on average 2.9 mmol/L and a median of 2 (interquartile range 1–3) nonshock organ failures were present on admission, indicative of end-organ dysfunction in the setting of hypotension.

Half (48%) of the included shock patients had a normal LVEF (> 40%) within 1 day after arrest. Pre-arrest LVEF was available in 54 patients, in whom it was highly correlated with the post-arrest measure (ρ = 0.69; *p* < 0.01) but on average slightly higher than that post arrest (pre-arrest vs post-arrest difference in LVEF 7 ± 16%; *p* < 0.01).

Patients in shock despite normal LVEF had lower peak troponin, were less likely to be diagnosed with ST-elevation myocardial infarction, and were less likely to undergo coronary angiography, coronary stenting, or receive an intraaortic balloon pump (Table 1).

While 43% of included patients survived to hospital discharge, only 22% had a favorable neurocognitive outcome at discharge. Additional patient characteristics are presented in Table 1.

### LVEF and neurocognitive outcome

**Fig. 2:**
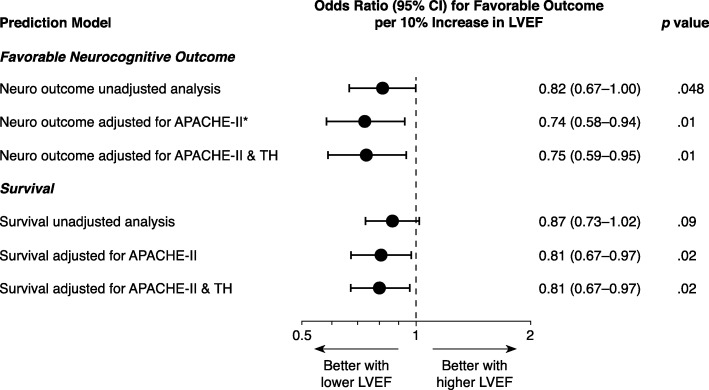
LVEF and patient outcomes after OHCA. Sensitivity analyses performed for favorable neurocognitive outcome (CPC 1–2, primary study outcome) and survival at hospital discharge, to confirm results were not dependent on covariate adjustment. ORs indicate odds of favorable vs unfavorable outcome per 10% increase in LVEF. Additional sensitivity analyses (see Additional file 1) confirmed results did not depend on method of quantifying illness severity nor handling of dependent and independent variables. *Prespecified primary analysis of main outcome. CI confidence interval, LVEF left ventricular ejection fraction, APACHE II Acute Physiological and Chronic Health Evaluation II, TH therapeutic hypothermia

In unadjusted analysis, higher LVEF was associated with less favorable neurocognitive outcome (OR for favorable neurocognitive outcome 0.82, 95% CI 0.67–1.00 per 10% increase in LVEF; *p* = 0.048) (Fig. 2).

**Fig. 3:**
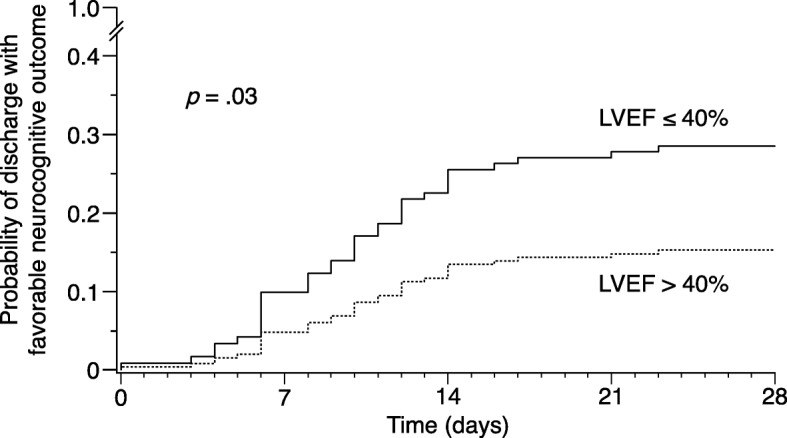
Adjusted probability of discharge with favorable neurocognitive outcome over time. Calculated from Cox proportional hazards model adjusting for APACHE II score. Hazard ratio for normal LVEF compared to low LVEF 0.46, 95% CI 0.23–0.91; *p* = 0.03. LVEF left ventricular ejection fraction

In the prespecified primary analysis, higher LVEF remained associated with less favorable neurocognitive outcome after adjusting for APACHE II score (OR 0.74, 95% CI 0.58–0.94 per 10% increase in LVEF; *p* = 0.01). Sensitivity analyses confirmed that the association between higher LVEF and less favorable neurocognitive outcome did not depend on the method of quantifying illness severity, the included covariates, or handling of the dependent and independent variables (Fig. 2; Additional file 1: Table S2). The linearity assumption of LVEF with favorable neurocognitive outcome on the log-odds scale held up on testing for the range of values in our dataset. The Cox APACHE II score-adjusted cumulative incidence for discharge with favorable neurocognitive outcome according to normal vs low LVEF is shown in Fig. 3; the Cox model proportionality assumption was tested and deemed valid.

In the expanded sensitivity cohort (*n* = 235), normal LVEF again was associated with less favorable neurocognitive outcome compared to low LVEF in unadjusted analysis (OR 0.48, 95% CI 0.25–0.90 for normal vs low LVEF; *p* = 0.02) and APACHE II score-adjusted analysis (OR 0.33, 95% CI 0.16–0.67; *p* < 0.01).

### LVEF and survival

In unadjusted analysis, higher LVEF was not significantly associated with survival to discharge (OR 0.87, 95% CI 0.73–1.02 per 10% increase in LVEF; *p* = 0.09). However, after adjusting for baseline illness severity via the APACHE II score, higher LVEF was significantly associated with less survival (OR for survival 0.81, 95% CI 0.67–0.97 per 10% increase in LVEF; *p* = 0.02). This association remained significant after adding therapeutic hypothermia to the APACHE II score-adjusted model (Fig. 2).

### LVEF and other secondary outcomes

**Fig. 4:**
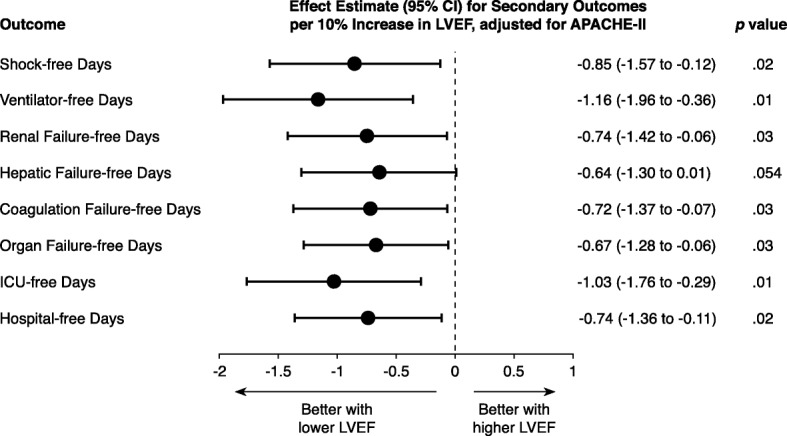
LVEF and secondary outcomes. Effect estimates with 95% CIs for outcome per 10% increase in LVEF from linear regression models adjusting for APACHE II score. CI confidence interval, LVEF left ventricular ejection fraction, APACHE II Acute Physiological and Chronic Health Evaluation II, ICU intensive care unit

Higher LVEF was associated with fewer days free from shock, mechanical ventilation, renal failure, coagulation failure, and any organ failure in APACHE II score-adjusted analyses (Fig. 4). LVEF was not associated with hepatic failure-free days. Higher LVEF also was associated with fewer ICU-free days and fewer hospital-free days in APACHE II score-adjusted analyses (Fig. 4).

### Subgroup analysis by initial rhythm

Among patients with an arrest rhythm of ventricular tachycardia or ventricular fibrillation (*n* = 86), the odds ratio for neurocognitive outcome favored lower LVEF, but this association did not reach statistical significance (OR 0.76, 95% CI 0.54–1.07; *p* = 0.10). Among patients with a nonshockable rhythm (*n* = 76), there was no suggestion of association between LVEF and favorable neurocognitive outcome (OR 0.94, 95% CI 0.59–1.50; *p* = 0.81).

In the expanded sensitivity cohort, normal LVEF was associated with less favorable neurocognitive outcome among patients with ventricular tachycardia or ventricular fibrillation (*n* = 108; OR 0.34, 95% CI 0.12–0.93; *p* = 0.03) and was not associated with neurocognitive outcome among patients with nonshockable rhythm (*n* = 127; OR 1.01, 95% CI 0.19–5.22; *p* = 1.00).

### Volume resuscitation and clinical outcomes

Intravascular volume resuscitation did not differ for patients with normal vs low LVEF during the first 6 h (2.9 ± 1.8 vs 2.7 ± 1.7 l; *p* = 0.504) nor through 24 h (6.1 ± 3.5 vs 5.6 ± 3.1 l; *p* = 0.34) after arrest. Only half of all shock patients (51%) received a volume challenge of at least 30 ml/kg body weight in the first 6 h post arrest.

Among patients with normal LVEF, greater intravascular volume resuscitation during the first 6 h post arrest was associated with favorable neurocognitive outcome (OR 1.59, 95% CI 0.99–2.55 per liter of volume; *p* = 0.03) and survival (OR 1.44, 95% CI 1.02–2.04; *p* = 0.02) in models adjusting for APACHE II score, baseline vasopressor dose, and arrest rhythm. By contrast, among patients with low LVEF, no association was found between 6-h volume resuscitation and either neurocognitive outcome or survival. Total 24-h volume resuscitation was not associated with neurocognitive outcome or survival irrespective of LVEF.

### Analyses for residual confounding

LVEF was associated with neither mean arterial pressure nor vasopressor dose, regardless of whether baseline or 48-h time-weighted average value was considered (see Additional file 1). Higher LVEF was associated with less use of therapeutic hypothermia (OR 0.80, 95% CI 0.64–1.00; *p* = 0.047), likely owing to differences in incidence of initial shockable rhythm among normal vs low LVEF patients (32% vs 73%, respectively; *p* < 0.01). However, adding therapeutic hypothermia to the models did not change the significant association between higher LVEF and less favorable clinical outcomes, and therapeutic hypothermia was not associated with favorable neurocognitive outcome or survival in this cohort.

## Discussion

PCAS is a state of acute end-organ injury stemming from intra-arrest global tissue hypoxia and subsequent ischemia–reperfusion injury, exacerbated by post-arrest hemodynamic instability, inflammation, and persistent effects of the underlying precipitant of arrest [7]. Clinical experience and existing literature indicate substantial physiological and biological heterogeneity among PCAS patients following OHCA [32, 33]. Recognition of clinically discernible phenotypes therefore may provide both prognostic and potential therapeutic value.

This study supports considering at least two subtypes of circulatory shock in PCAS: cardiogenic-predominant shock and distributive-predominant (noncardiogenic) shock. In this study, the more shock reflected distributive rather than cardiogenic physiology, the worse the patient’s outcome. Among patients in post-resuscitation shock after OHCA, higher LVEF was associated with worse neurocognitive outcome. This conclusion held through several sensitivity analyses scrutinizing covariate adjustment and handling of the independent and dependent variables of interest. Higher LVEF also was associated with prolonged duration of shock and organ failures and greater risk of death among patients in post-resuscitation shock.

In addition to prognostic value, this study also suggests that subtyping PCAS shock could have potential relevance to personalizing treatment strategies. For instance, a patient with cardiogenic shock typically would not receive early aggressive intravascular volume resuscitation, which by contrast is standard of care for inflammation-mediated distributive shock, such as in sepsis [34–37].

In our cohort, only half (51%) of the patients received a volume challenge ≥ 30 ml/kg in the first 6 h post arrest, and cumulative volume resuscitation during this period did not differ by shock subtype. Intravascular volume resuscitation exhibited a subtype-specific association with clinical outcomes: more volume resuscitation during the first 6 h post arrest was associated with better neurocognitive outcome and survival among patients in shock with normal LVEF, but no such association was found among patients in shock with low LVEF. Volume received through 24 h was not predictive of outcome in either subtype. Thus, as with sepsis, these data suggest—but do not prove—that volume resuscitation may be most effective early in the course of proinflammatory distributive shock.

Several possibilities may explain the observed link between distributive-predominant shock physiology and worse outcome. The natural history of cardiogenic shock may be more favorable in the subset of patients in whom myocardial stunning is the primary contributor because severe cardiac dysfunction can be transient in these patients [1, 10]. Supporting this possibility, lower LVEF was associated with more shock-free days in our cohort. In contrast, with distributive shock, systemic inflammation may be propagated for days by the immune response to damage-associated and pathogen-associated molecular patterns, contributing to ongoing end-organ injury long after return of spontaneous circulation [16]. This underlying biology may explain the association of higher LVEF with longer duration of multiple organ failures and worse neurocognitive outcome in this cohort.

Low LVEF is a known risk factor for arrest due to ventricular arrhythmia, which typically is associated with better prognosis [38]. Thus, it is possible that the association between lower LVEF and better outcomes is explained in part by arrest etiology. However, a similar trend linking higher LVEF to poorer outcome was found in subgroup analysis restricted to patients with ventricular arrhythmia.

Other baseline differences may exist between patients that were not adequately measured or addressed in this study. Yet typical measures of overall illness severity (APACHE II score, SOFA score, number of organ failures) did not differ by normal vs low LVEF, and analyses adjusted for potential baseline differences. LVEF was not associated with initial vasopressor dose, excluding baseline shock severity as a confounder. Still, cardiac function may be augmented by infusion of vasopressors that have concomitant inotropic effects, including norepinephrine [39], complicating attribution of shock subtype by LVEF without ancillary supportive data.

Therapeutic hypothermia was more commonly used in patients with LVEF ≤ 40% due to their higher incidence of ventricular arrhythmia. Yet hypothermia was not associated with neurocognitive outcome or survival in this cohort, and including hypothermia as a model covariate did not change the conclusions. LVEF was not associated with mean arterial pressure at baseline or through 48 h, such that differences in cerebral perfusion pressure are unlikely to explain this association.

The present study used LVEF to infer underlying shock physiology. The timing of LVEF assessment was restricted to within 1 day of arrest for main analyses to ensure transient myocardial stunning was accurately identified.

LVEF alone is an imperfect surrogate of cardiac output, although the likelihood that severely depressed systolic function signifies cardiogenic pathophysiology is high in the setting of post-arrest shock [1]. Future studies should incorporate repeated measures of cardiac function, systemic vascular resistance, and inflammatory markers to better delineate shock physiology.

Overlap in hemodynamic features and underlying biology undoubtedly exists among PCAS shock subtypes, but such is not unique to the post-arrest patient. Cardiogenic shock from acute coronary syndrome can cause concomitant systemic inflammation, which in turn may exacerbate myocardial dysfunction and impair peripheral vascular compensation [40–42]. In septic shock, inflammation-induced myocardial depression, microcirculatory derangements, and autonomic dysfunction contribute to cardiac dysfunction [43–45]. Other classic shock subtypes—hypovolemic and obstructive shock—could also occur post arrest depending on underlying precipitant of arrest. Despite this overlap, discerning the predominant shock physiology may help guide prognostication and therapeutic decision-making. Still, causation cannot be inferred from this cohort study. Clinical trials will be required to delineate treatment implications, including the role, if any, for early aggressive volume resuscitation in PCAS shock subtypes.

## Conclusions

Distributive-predominant (noncardiogenic) shock—as reflected by higher LVEF—was associated with worse neurocognitive outcome, prolonged organ failures, and higher mortality among patients in PCAS shock. Greater volume resuscitation in the first 6 h post arrest was associated with more favorable neurocognitive outcome and survival among patients with distributive but not cardiogenic shock physiology. Future studies with repeated measures of complementary hemodynamic parameters are needed to independently validate the prognostic and therapeutic value for subtyping PCAS shock.

## Additional file

Additional file 1: Supplemental material including additional details on methods, supplemental results, and Tables S1 and S2. (PDF 161 kb)

## Abbreviations

APACHE II: Acute Physiology and Chronic Health Evaluation II
CI: Confidence interval
CPC: Cerebral Performance Category
ICU: Intensive care unit
LVEF: Left ventricular ejection fraction
OHCA: Out-of-hospital cardiac arrest
OR: Odds ratio
PCAS: Post-cardiac arrest syndrome
SOFA: Sequential Organ Failure Assessment
