# Supplementary material for: Shock subtypes by left ventricular ejection fraction following out-of-hospital cardiac arrest
Source: Crit Care. 2018 Jun 15;22:162. doi: 10.1186/s13054-018-2078-x (PMC6003130; doi:10.1186/s13054-018-2078-x)
Supplement: Supplementary file 1 — Supplemental material including additional details on methods, supplemental results, and Tables S1 and S2. (PDF 161 kb) [file 13054_2018_2078_MOESM1_ESM.pdf]

Shock Subtypes by Left Ventricular Ejection Fraction Following Out-of-Hospital Cardiac Arrest

Anderson RJ, Jinadasa SP, Hsu L, Ghafouri TB, Tyagi S, Joshua J, Mueller A, Talmor D, Sell RE, Beitler JR

SUPPLEMENTAL MATERIAL

ADDITIONAL DETAILS ON METHODS

Hemodynamic Resuscitation

Vasopressor requirements were reported in norepinephrine-equivalent dose (μg/min), calculated as [norepinephrine (μg/min)] + [epinephrine (μg/min)] + [dopamine (μg/kg/min) × 0.5] + [phenylephrine (μg/min) × 0.1] + [vasopressin (units/min) × 5/0.03], in similar fashion to the VASST trial [1].

Volume resuscitation in the first six and first 24 hours was recorded with time zero beginning at arrival in the emergency department, to which was added any pre-hospital volume administered by emergency medical services. All documented volumes of intravenous or intraosseous infusions of crystalloid, colloid, and blood products were included. When blood products were charted only in units, we assumed a volume of 300 mL per unit red blood cell transfusion and 250 mL per unit fresh frozen plasma or pooled platelet bag transfusion. If continuous renal replacement therapy was initiated, only the net fluid balance was considered during that time.

Peri-Arrest Characteristics and Management

Other peri-arrest characteristics were obtained by reviewing pre-hospital first-responder, emergency department, air ambulance (if inter-hospital transfer), catheterization lab, and inpatient flow sheets and physician, nurse, and technician notes. Cardiac arrest data were extracted following Utstein template recommendations [2]. Additional in-hospital data extracted included illness severity scores (Acute Physiology and Chronic Health Evaluation-II [APACHE-II] [3], Sequential Organ Failure Assessment [SOFA] [4,5], Brussels definitions of organ failures for coagulation, renal, and hepatic organ systems [6-8]), clinical diagnosis of ST-elevation myocardial infarction, cardiac catheterization and coronary stenting, respiratory, and laboratory data.

Ascertainment of Cerebral Performance Category (CPC)

Each evaluator for CPC was provided the following detailed criteria, quoted directly from the Brain Resuscitation Clinical Trial I Study Group [9,10], to ascertain CPC for each patient:

1. **CPC 1 – Good Cerebral Performance (*Normal life*):** Conscious, alert, able to work and lead a normal life. May have minor psychological or neurologic deficits (mild dysphasia, non-incapacitating hemiparesis, or minor cranial nerve abnormalities).

2. **CPC 2 – Moderate Cerebral Disability (*Disabled but independent*):** Conscious with sufficient cerebral function for part-time work in sheltered environment or independent activities of daily life (dress, travel by public transportation, food preparation). May have hemiplegia, seizures, ataxia, dysarthria, dysphasia, or permanent memory or mental changes.
3. **CPC 3 – Severe Cerebral Disability (*Conscious but disabled and dependent*):** Conscious but dependent on others for daily support (in an institution or at home with exceptional family effort). Has at least limited cognition. This category includes a wide range of cerebral abnormalities, from patients who are ambulatory but have severe memory disturbances or dementia precluding independent existence to those who are paralyzed and can communicate only with their eyes, as in the locked-in syndrome.
4. **CPC 4 – Coma / Vegetative State (*Unconscious*):** Unconscious, unaware of surroundings, no cognition. No verbal or psychological interaction with environment.
5. **CPC 5 – Brain Death (*Certified brain dead or dead by traditional criteria*):** Certified brain death or dead by traditional criteria.

CPC on hospital discharge was determined retrospectively by reviewing each patient's medical record as previously described [11]. As available, evaluators reviewed the discharge summary, discharge referral form, and the last documented notes from the primary treatment team, neurology service, nursing, physical and occupational therapy, social worker, and case manager. Evaluations were performed blinded to left ventricular ejection fraction, hemodynamic and resuscitation data, and other illness severity measures. Two investigators, (1) a dedicated site-specific investigator for each site and (2) a study-wide investigator with access to all records at both sites, independently reviewed each chart, with discordant ratings resolved by consensus for the final dataset.

The primary outcome, specified a priori, was favorable neurocognitive outcome at hospital discharge, defined as CPC of 1 or 2. We chose to dichotomize CPC for our primary analysis to facilitate ease of understanding results, as has been done in recent high-profile cardiac arrest clinical trials [12-14]. To ensure results were not dependent on dichotomizing CPC, data were re-analyzed using ordinal logistic regression with CPC entered as an ordinal dependent variable.

Organ Failure-Free Days

Secondary outcomes included shock-free days, ventilator-free days, and renal, hepatic, and coagulation failure-free days. Organ failure-free days were calculated as the number of days between sustained resolution of organ failure and day 28 [8,11,15]. If a patient temporarily recovered but developed organ failure again later in the hospital course, the period of transient recovery did not count toward failure-free days. When data were missing for a given day, the last observed value was carried forward until day of live hospital discharge. Organ failures were considered resolved following live hospital discharge, except for renal failure in patients scheduled to undergo post-discharge dialysis. A value of zero failure-free days was assigned if the patient died before hospital discharge. ICU-free days and hospital-free days were also evaluated and calculated similarly.

Except as noted, organ failures were determined by applying Brussels Multiple Organ Dysfunction consensus conference definitions for clinically significant organ dysfunction [6].

Shock was defined as systolic blood pressure ≤ 90 mmHg or any vasopressor use. Pulmonary failure was defined as receipt of invasive mechanical ventilation. Renal failure was defined as creatinine ≥ 2.0 mg/dL or receipt of renal replacement therapy. Hepatic failure was defined as total bilirubin ≥ 2 mg/dL. Coagulation failure was defined as platelet count ≤ 80 x $10^3/mm^3$. The worst values for each calendar day were used in determining if organ failure was present on a given day. Patients receiving chronic outpatient dialysis prior to admission were excluded from analysis of renal failure-free days.

Sensitivity Analyses for Primary Outcome

Multiple sensitivity analyses were performed for the primary outcome, favorable neurocognitive outcome, to ensure findings were not dependent on method of covariate adjustment or handling of the main predictor or outcome variables. Alternative covariate adjustments included replacing APACHE-II with either SOFA or number of organ failures at baseline, and adding therapeutic hypothermia as a covariate. To better reflect clinical decision-making and determine if results were dependent on handling LVEF as a continuous variable, LVEF was re-entered as a dichotomized variable (normal LVEF > 40% vs. low LVEF ≤ 40%) in an APACHE-II-adjusted model. In another sensitivity analysis, CPC was entered as an ordinal outcome analyzed via ordinal logistic regression. Finally, the main analysis was repeated using the expanded sensitivity cohort described above.

ADDITIONAL RESULTS

Baseline Characteristics

Baseline characteristics for patients not in shock are presented in **Table S1**, accompanied by included patient characteristics for comparison.

Sensitivity Analyses for Primary Endpoint

Multiple sensitivity analyses confirmed that the association between higher LVEF and less favorable neurocognitive outcome did not depend on method of quantifying illness severity, included covariates, or handling of the dependent and independent variables (**Figure 2**; **Table S2**). Adjusting for alternative measures of illness severity (Day 1 SOFA, number of organ failures at baseline) again demonstrated the association between higher LVEF and less favorable neurocognitive outcome. Adding therapeutic hypothermia as a covariate in these models also did not change this association, and in no model was therapeutic hypothermia associated with neurocognitive outcome.

Reanalysis entering LVEF as a dichotomized variable and adjusting for APACHE-II similarly found that normal LVEF, compared to low LVEF, was associated with less favorable neurocognitive outcome (OR 0.36, 95% CI 0.15-0.85; p = .02). Reanalysis using ordinal logistic regression, with CPC entered as the outcome variable and adjusting for APACHE-II, confirmed that the association between higher LVEF and less favorable neurocognitive outcome was not dependent on dichotomizing CPC (OR 0.80, 95% CI 0.67-0.95 for 1-unit change in CPC per 10% increase in LVEF; p = .01; score test for proportional odds assumption p = .31).

Sensitivity analyses also were performed to address the 73 patients in shock excluded from the main analyses for lack of an LVEF measurement within one calendar day of arrest (**Figure 1; Table 1**). The expanded sensitivity cohort (n=235) considered LVEF assessment any time during admission and assumed that patients without an LVEF assessment during admission had normal LVEF. In this expanded cohort, normal LVEF was associated with less favorable neurocognitive outcome compared to low LVEF in unadjusted analysis (OR 0.48, 95% CI 0.25-0.90 for normal vs. low LVEF; $p = .02$) and APACHE-II-adjusted analysis (OR 0.33, 95% CI 0.16-0.67; $p < .01$).

Analyses for Residual Confounding

LVEF was not associated with mean arterial pressure at baseline (ß = -0.07, 95% CI -1.68, 1.53 for mmHg change in MAP per 10% increase in LVEF; $p = .93$) nor with time-weighted average mean arterial pressure over the first 48 hours (ß = 0.49, 95% CI -0.24, 1.22; $p = .19$).

Similarly, LVEF was not associated with baseline vasopressor dose (ß = -0.05, 95% CI -1.88, 1.78 for µg/min change in norepinephrine-equivalent rate per 10% increase in LVEF; $p = .96$) nor time-weighted average vasopressor dose over the first 48 hours (ß = 0.21, 95% CI -0.78, 1.21; $p = .67$).

Table S1. Characteristics among included patients compared to those without shock but otherwise eligible

Patient Characteristic	Shock with LVEF > 40% (n = 78)	Shock with LVEF ≤ 40% (n = 84)	*p*	No Shock (n = 70)
Age (years)	63 ± 15	64 ± 15	.73	60 ± 19
Female	26 (33%)	20 (24%)	.22	22 (31%)
Comorbidities				
Coronary disease	18 (23%)	34 (40%)	.02	23 (33%)
Congestive heart failure	12 (15%)	34 (40%)	< .01	15 (21%)†
Chronic pulmonary disease	12 (15%)	11 (13%)	.82	14 (20%)
Arrest characteristics				
Witnessed arrest	56 (72%)	68 (81%)	.20	56 (80%)
Bystander CPR	41 (53%)	50 (60%)	.43	41 (59%)
Time from collapse to CPR initiation (min)	2 [1—7]	2 [0—6]	.97	1 [0—5]
Duration of CPR before sustained ROSC (min)	15 [10—38]	18 [9—30]	.65	15 [7—25]
Initial rhythm VT/ VF	25 (32%)	61 (73%)	< .01	42 (60%)*
Comatose after ROSC	72 (92%)	80 (95%)	.52	59 (84%)†‡
Therapeutic hypothermia after ROSC	59 (76%)	75 (89%)	.02	51 (73%)†
Cardiac characteristics				
Peak troponin in first 24h (ng/mL)	0.3 [0.1—1.0]	0.9 [0.3—2.7]	< .01	0.3 [0.1—0.9]†
ST-elevation MI	8 (10%)	23 (27%)	.01	14 (20%)
Coronary stent placed during hospitalization	7 (9%)	20 (24%)	.01	15 (21%)*
LVEF (%) within ≤ 1 day after arrest	59 ± 10	26 ± 9	< .01	45 ± 17*†
Markers of systemic illness severity				
APACHE-II	35 ± 6	36 ± 6	.09	31 ± 6*†‡
SOFA on Day 1	12 ± 3	12 ± 3	.67	8 ± 2*†‡
Number of organ failures on day 1§	3 ± 1	3 ± 1	.97	2 ± 1*†‡
Initial lactate (mmol/L)	5.8 ± 4.7	4.1 ± 2.8	.01	4.8 ± 5.8
Peak lactate in first 24h (mmol/L)	5.9 ± 4.7	4.6 ± 3.1	.04	4.5 ± 5.3
Initial respiratory characteristics				
Tidal volume (mL/kg PBW)	8.0 ± 1.9	8.1 ± 1.8	.79	8.0 ± 1.4
PEEP (cmH_2O)	5 [5—8]	5 [5—10]	.14	5 [5—5]†
Peak inspiratory pressure (cmH_2O)	27 ± 8	26 ± 8	.75	25 ± 7
FiO_2	100 [80—100]	100 [60—100]	.40	100 [50—100]
pH	7.22 ± 0.19	7.23 ± 0.15	.53	7.29 ± 0.15*†‡
$PaCO_2$ (mmHg)	44 [39—62]	47 [37—56]	.66	40 [36—48]*†‡
PaO_2 (mmHg)	212 ± 128	202 ± 130	.62	255 ± 160†‡
PaO_2:FiO_2	237 ± 141	237 ± 148	.99	290 ± 185‡
Volume resuscitation in first 6h (liters)	2.9 ± 1.8	2.7 ± 1.7	.50	2.0 ± 1.2*†‡
Volume challenge ≥ 30 mL/kg in first 6h	42 (54%)	41 (49%)	.53	22 (31%)*†‡
Volume resuscitation in first 24h (liters)	6.1 ± 3.5	5.6 ± 3.1	.34	4.0 ± 2.0*†‡

* p-value < .05 compared to patients with LVEF > 40%.
† p-value < .05 compared to patients with LVEF ≤ 40%
‡ p-value < .05 compared to all included patients with LVEF assessment within ≤ 1 day post-arrest
§ Cardiovascular failure was defined as systolic blood pressure ≤ 90 mmHg or any vasopressor use. Respiratory failure was defined by invasive mechanical ventilation. Coagulation, renal, and hepatic organ failures were defined according to the Brussels multiple organ dysfunction criteria.[6-8]
Abbreviations: LVEF, left ventricular ejection fraction; CPR, cardiopulmonary resuscitation; ROSC, return of spontaneous circulation; VT, ventricular tachycardia; VF, ventricular fibrillation; APACHE-II, Acute Physiology and Chronic Health Evaluation-II score; SOFA, sequential organ failure assessment score; PEEP, positive end-expiratory pressure.

Table S2: Sensitivity analysis logistic regression models of odds ratio for favorable neurocognitive outcome per 10% increase in LVEF

Models predicting favorable neurocognitive outcome	OR (95% CI)	*p*
LVEF	0.82 (0.67-1.00)	.048
LVEF + APACHE-II	0.74 (0.58-0.94)	.01
LVEF + $SOFA_{DAY1}$	0.76 (0.60-0.95)	.01
LVEF + $\text{Organ-failures}_{DAY1}$	0.80 (0.64-0.99)	.03
LVEF + APACHE-II + TH	0.75 (0.59-0.95)	.01
LVEF + $SOFA_{DAY1}$ + TH	0.76 (0.60-0.95)	.01
LVEF + $\text{Organ-failures}_{DAY1}$ + TH	0.78 (0.63-0.98)	.03

Abbreviations: LVEF, left ventricular ejection fraction; APACHE-II, Acute Physiology and Chronic Health Evaluation-II; SOFA, sequential organ failure assessment; TH, therapeutic hypothermia.

SUPPLEMENT REFERENCES

1. Russell JA, Walley KR, Singer J, Gordon AC, Hébert PC, Cooper DJ, et al. Vasopressin versus norepinephrine infusion in patients with septic shock. N. Engl. J. Med. 2008;358:877–87.

2. Jacobs I, Nadkarni V, Bahr J, Berg RA, Billi JE, Bossaert L, et al. Cardiac arrest and cardiopulmonary resuscitation outcome reports: update and simplification of the Utstein templates for resuscitation registries: a statement for healthcare professionals from a task force of the International Liaison Committee on Resuscitation (American Heart Association, European Resuscitation Council, Australian Resuscitation Council, New Zealand Resuscitation Council, Heart and Stroke Foundation of Canada, InterAmerican Heart Foundation, Resuscitation Councils of Southern Africa). Circulation. 2004;110:3385–97.

3. Knaus WA, Draper EA, Wagner DP, Zimmerman JE. APACHE II: a severity of disease classification system. Crit Care Med. 1985;13:818–29.

4. Vincent JL, Moreno R, Takala J, Willatts S, De Mendonça A, Bruining H, et al. The SOFA (Sepsis-related Organ Failure Assessment) score to describe organ dysfunction/failure. On behalf of the Working Group on Sepsis-Related Problems of the European Society of Intensive Care Medicine. Intensive Care Med. 1996;22:707–10.

5. Vincent JL, De Mendonça A, Cantraine F, Moreno R, Takala J, Suter PM, et al. Use of the SOFA score to assess the incidence of organ dysfunction/failure in intensive care units: results of a multicenter, prospective study. Working group on “sepsis-related problems” of the European Society of Intensive Care Medicine. Crit Care Med. 1998;26:1793–800.

6. Bernard G. The Brussels score. Sepsis. Springer; 1997;1:43–4.

7. Bernard GR. Quantification of organ dysfunction: seeking standardization. Crit Care Med. 1998;26:1767–8.

8. Acute Respiratory Distress Syndrome Network. Ventilation with lower tidal volumes as compared with traditional tidal volumes for acute lung injury and the acute respiratory distress syndrome. N. Engl. J. Med. 2000;342:1301–8.

9. Brain Resuscitation Clinical Trial I Study Group. A randomized clinical study of cardiopulmonary-cerebral resuscitation: design, methods, and patient characteristics. Am J Emerg Med. 1986;4:72–86.

10. Brain Resuscitation Clinical Trial I Study Group. Randomized clinical study of thiopental loading in comatose survivors of cardiac arrest. N. Engl. J. Med. 1986;314:397–403.

11. Beitler JR, Ghafouri TB, Jinadasa SP, Mueller A, Hsu L, Anderson RJ, et al. Favorable neurocognitive outcome with low tidal volume ventilation after cardiac arrest. Am. J. Respir. Crit. Care Med. 2017;195:1198–206.

12. Nielsen N, Wetterslev J, Cronberg T, Erlinge D, Gasche Y, Hassager C, et al. Targeted Temperature Management at 33°C versus 36°C after Cardiac Arrest. N. Engl. J. Med. 2013;369:2197–206.

13. Hypothermia after Cardiac Arrest Study Group. Mild therapeutic hypothermia to improve the neurologic outcome after cardiac arrest. N. Engl. J. Med. 2002;346:549–56.

14. Mentzelopoulos SD, Malachias S, Chamos C, Konstantopoulos D, Ntaidou T, Papastylianou A, et al. Vasopressin, steroids, and epinephrine and neurologically favorable survival after in-hospital cardiac arrest: a randomized clinical trial. JAMA. 2013;310:270–9.

15. Schoenfeld DA, Bernard GR, ARDS Network. Statistical evaluation of ventilator-free days as an efficacy measure in clinical trials of treatments for acute respiratory distress syndrome. Crit Care Med. 2002;30:1772–7.
